# Cervical Multifidus and Longus Colli Ultrasound Differences among Patients with Cervical Disc Bulging, Protrusion and Extrusion and Asymptomatic Controls: A Cross-Sectional Study

**DOI:** 10.3390/jcm13020624

**Published:** 2024-01-22

**Authors:** Khodabakhsh Javanshir, Payam Ghafouri-Rouzbehani, Amirhossein Zohrehvand, Arvin Naeimi, César Fernández-de-las-Peñas, Hossein-Ali Nikbakht, Seyedeh Roghayeh Mousavi-Khatir, Juan Antonio Valera-Calero

**Affiliations:** 1 Department of Physical Therapy, School of Rehabilitation, Babol University of Medical Science, Babol 47176-47745, Iran; khodabakhshjavanshir@gmail.com (K.J.); payam.ghafouri@gmail.com (P.G.-R.); rmosavi_pt@yahoo.com (S.R.M.-K.); 2 Department of Neurosurgery, School of Medicine, Babol University of Medical Sciences, Babol 47176-47745, Iran; ahz59dr@gmail.com; 3 Student Research Committee, School of Medicine, Guilan University of Medical Sciences, Rasht 41446-66949, Iran; arvin.naeimi@gmail.com; 4 Department of Physical Therapy, Occupational Therapy, Physical Medicine and Rehabilitation, Universidad Rey Juan Carlos, 28922 Madrid, Spain; cesar.fernandez@urjc.es; 5 Social Determinants of Health Research Center, Health Research Institute, Babol University of Medical Sciences, Babol 47176-47745, Iran; ep.nikbakht@gmail.com; 6 Department of Radiology, Rehabilitation and Physiotherapy, Faculty of Nursery, Physiotherapy and Podiatry, Complutense University of Madrid, 28040 Madrid, Spain; 7 Grupo InPhysio, Instituto de Investigación Sanitaria del Hospital Clínico San Carlos (IdISSC), 28040 Madrid, Spain

**Keywords:** disc, neck pain, ultrasound, cervical multifidus, longus colli

## Abstract

The aim of this study was to analyze the differences in morphological and histological features of the cervical multifidus (CM) and longus colli (LC) muscles among patients with cervical disc bulging, protrusion, or extrusion. Fifteen patients with cervical disc bulging (20% male, mean age: 48.5, standard deviation (SD) 7.5 years), fifteen with cervical disc protrusion (6% male, mean age: 43, SD 7.8 years), and fifteen with cervical disc extrusion (40% male, mean age: 44, SD 8 years) diagnosed via clinical and imaging findings participated in this study. Additionally, fifteen asymptomatic controls (40% male, mean age: 40.4, SD 9.7 years) were also included. The following ultrasound measurements, cross-sectional area (CSA), anterior–posterior distance (APD), lateral dimension (LD), and mean echo-intensity (EI) of the CM and LC at C5-C6 level were examined by an assessor blinded to the subject’s condition. The results revealed no group ×side significant differences among the groups (*p* > 0. 00625). However, group effects were found for APD and MEI of the CM (*p* = 0.006 and *p* < 0.001, respectively) and CSA, APD and MEI of the LC (all, *p* < 0.001). The LD of the LC muscle and the APD and LD of the CM were negatively associated with related disability (*p* < 0.01; *p* < 0.05 and *p* < 0.01, respectively), and pain intensity was negatively associated with LC APD and LD (both *p* < 0.05). These results suggest that US can be used to detect bilateral morphological changes in deep cervical flexors and extensors to discriminate patients with cervical disc alterations.

## 1. Introduction

The cervical multifidus (CM), a deep neck extensor muscle located at the posterior aspect of the neck, plays a relevant role in the biomechanics and functional stability of the cervical spine [1]. Its anatomy and function have been extensively described in the literature, emphasizing its significance in segmental stabilization of the cervical spine [2]. The CM is divided into superficial and deep fascicles and runs the facet capsule of C4-C7 segments to the spinous process of C2 [2].

From a clinical perspective, the morphology and histology of the CM have been correlated with several neck pain conditions, including whiplash-associated disorders (WADs) [3], chronic idiopathic neck pain [4], cervical radiculopathy [5], cervical spondylosis [6] or spondylotic myelopathy [7,8]. For instance, Abbott et al. [3] described increased intramuscular fatty infiltration within the CM muscle in patients with chronic WAD as compared to recovered patients and pain-free healthy controls. A meta-analysis by Owers et al. [9] investigating morphometric changes in cervical muscles after WAD concluded that the cross-sectional area (CSA) of the CM was generally higher in individuals with WADs (particularly at the C5-C6 level) compared to pain-free subjects, although the results were heterogeneous. Considering that the morphological and histological changes in the CM are suggested to lead to impaired sensorimotor function, poorer postural stability, and increased disability [10,11], scientific evidence strongly advocates for a comprehensive analysis of these metrics during the initial evaluations of patients with neck pain [12]. Such an assessment not only provides a baseline understanding of the patient’s condition but also aids in tailoring therapeutic interventions focusing on therapeutic exercise programs. Furthermore, periodic reassessments during follow-ups become crucial to determine the efficacy of the treatment modalities employed and to make necessary adjustments [13].

Morphological findings in individuals with chronic non-specific neck pain remain controversial, and no firm conclusions can be made yet [14,15]. For instance, while some studies did not report either shape or size differences within CM between patients with chronic non-specific neck pain and healthy controls [14], other authors have described that patients with neck pain are characterized by smaller CSA at different levels of CM compared to the controls [16]. Thus, others described the opposite findings (larger CSA in patients with chronic neck pain than in asymptomatic subjects) [17].

The longus colli (LC) muscle has also been a muscle of interest in patients with neck pain. In fact, the literature describes more consistent findings for morphological changes within deep neck flexors than deep neck extensors [18]. For instance, Javanshir et al. [19] found that patients with mechanical bilateral chronic neck pain exhibited smaller bilateral CSA and anteroposterior (AP) distance of the LC compared with controls. These findings seem to be more present on the symptomatic side and correlated with clinical features such as neck pain intensity and duration or related disability [19,20], but not associated with the ability of the patient to perform deep neck flexor contraction (without the activation of superficial muscles) during a low-load task such as cranio–cervical flexion tests [21]. Although findings concerning other pain cohorts, such as cervical radicular pain [22,23], were comparable, neck pain etiology seems to play a relevant role in ultrasound (US) findings as specific populations, such as patients with cervicogenic headache, showed no differences with the healthy controls [24].

One important limitation of clinical practice is that several published studies have used magnetic resonance imaging (MRI), which is not readily accessible for physiotherapists and other clinicians due to the expensive costs in terms of human and material resources [25]. Ultrasound imaging (US) has emerged as a modality for assessing cervical muscles due to its advantages over other imaging techniques like MRI, CT, or X-ray. Thus, US offers real-time information, and it is cost-effective, faster, safer, and more accessible [26]. Recent advancements in US technology have further enhanced its diagnostic capabilities, allowing for the measurement of muscle metrics, including morphology and muscle histology [27,28], even in large muscles using a panoramic view [29]. In addition, the assessment of echo intensity (EI) is considered a valuable metric that can provide important histological information about the muscles, such as adipose tissue and/or connective tissue, which present with bright pixels while muscle fibers are hypoechoic [30]. Therefore, muscles with greater mean brightness would be characterized by a greater presence of fatty infiltration [31]. In addition, there are specific reliable procedures to reduce bias associated with US settings (as there is a high inter- and intra-subject variance due to the acoustic impedance variance), which uses relative measures to estimate the percentage of fatty infiltration within a muscle in different populations [18,32,33].

Since one important limitation recognized in previous studies assessing patients with neck pain is the absence of different groups regarding other radiological findings that could potentially influence the morphology and histology of these muscles, there is a need to investigate whether CM and LC muscles in patients with cervical disc bulging, protrusion, or extrusion may differ depending on their radiological findings when compared to asymptomatic controls. Thus, the primary aim of the current study was to analyze the differences in morphological and histological features of CM and LC muscles among asymptomatic controls and patients with cervical disc bulging, protrusion, or extrusion. We hypothesized that individuals with disc extrusion will exhibit greater atrophy and echo intensity in the CM and LC muscles when compared with those with disc bulging or protrusion. The secondary aims of this study were to compare the side-to-side differences among the groups and to analyze the association between demographic and clinical variables with US morphological features in these populations.

## 2. Methods

### 2.1. Study Design

A cross-sectional observational study investigating the differences in CM and LC morphology and brightness among individuals with different levels of cervical disc alterations (i.e., bulging, protrusion, or extrusion) and one cohort of asymptomatic subjects without radiological findings was conducted. This study followed the Strengthening the Reporting of Observational studies in Epidemiology (STROBE) guidelines and checklist [34]. Additionally, the authors considered all the recommendations disclosed in the Declaration of Helsinki to ensure the rights of the participants. The study design was approved by the local Clinical Ethics Committee of Babol University of Medical Sciences (IR.MUBABOL.REC.1401.144). All participants signed a written informed consent prior to data collection.

### 2.2. Participants

Patients with unilateral upper extremity pain admitted to Omid and Yahyanezhad medical centers, affiliated medical clinics of Babol University of Medical Sciences, Iran, from December 2022 to May 2023 were recruited. An experienced spine surgeon examined all patients, and those with a positive magnetic resonance imaging (MRI) finding confirmative for correlative posterolateral disc herniation (e.g., bulging, protrusion, and extrusion) at the level of C5-C6 were evaluated for eligibility criteria. Finally, patients between the ages of 20 and 60 years old with unilateral chronic radicular neck pain lasting for at least 12 weeks, accompanied by weakness in shoulder flexion, wrist extension, and a positive Spurling, were included in the study. The exclusion criteria consisted of the following: (1) cervical disc herniation at any level different than C5-C6; (2) history of spinal abnormality (e.g., spinal stenosis), spinal surgery, whiplash injury, cervical fracture, spinal deformity; (3) exercise training or physical therapy for the cervical spine within the past six months; (4) history of rheumatologic disease, neurologic disease, metabolic disorder, or malignancy; (5) allergic reaction to ultrasound gel; (6) lack of consent to participate in the study; (7) pregnancy or (8) any shoulder, elbow or wrist disorder (such as frozen shoulder, rotator cuff damage, or diseases of the muscle attachments around the elbow joint) as it could explain the upper limb weakness [35]. According to the degree of disc herniation, patients were then categorized into bulging, protrusion, and extrusion groups based on the MRI findings [36].

### 2.3. Sample Size Calculation

We calculated the sample size to detect between-group (4 groups) and within-group (2 sides) differences. An a priori analysis with an ANOVA test was conducted using the G*Power software v.3.1 (Dusseldorf, Germany), and the following input parameters were used: α = 0.05, β = 0.05 (95% power). According to the criteria established by Cohen [37], an effect size of small-to-moderate magnitude (0.3) was fixed, which was required to detect clinically relevant differences. Due to the cross-sectional nature of the study, no losses were expected. Therefore, this calculation led to a sample size of at least 52 participants (*n* = 13 per group).

### 2.4. Outcomes

All US images were acquired by the same experienced examiner with real-time B-mode US equipment (Samsung Medison US, SonoAce R7 system, Seoul, Republic of Korea) and a 5–12 MHz linear probe (LN5). All US settings were established as follows: 12.0 MHz, 55 dB for gain, and 3.5 cm for depth. Three images were taken for each muscle, with a time interval of one hour between each acquisition, and the mean of the three measurements was used to determine the muscle size used in the main analyses.

#### 2.4.1. Cervical Multifidus and Longus Colli Imaging Acquisition

**Figure 1:**
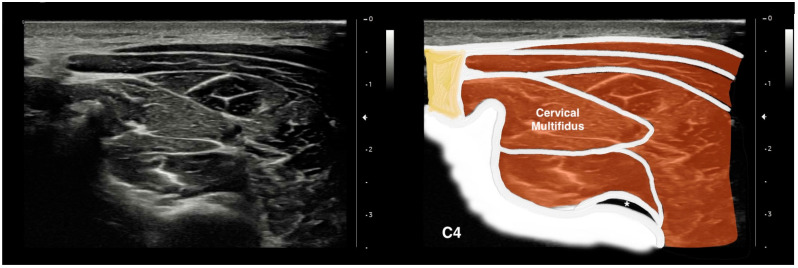
Ultrasound imaging of the cervical multifidus muscle at the C4 level. *: C4/C5 zygapophyseal joint; yellow: Nuchal ligament.

To image the CM muscle, participants sat on a chair with their head and neck in a neutral position. Keeping the nasal tip, chin, and sternal notch in a vertical line, the nasal base and occipital bone were horizontally aligned [38]. Participants were then requested to relax their cervical and shoulder muscles. Palpation was then performed to identify the spinous process of the C4 vertebra [39]. To measure the dimensions of the CM muscle, the ultrasound probe was moved horizontally and laterally on the posterior surface of the neck using a warm gel [40]. Upon clearly observing vertebral laminae and separating fascia, the image was frozen and saved for posterior analysis (Figure 1).

**Figure 2:**
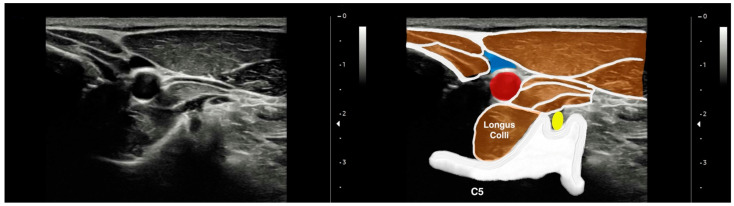
Ultrasound imaging of the longus colli muscle at the C5 level. Yellow: C4 root; blue: interior jugular vein; red: common carotid artery.

To image the LC muscle, the participants were placed in a supine position with their hands resting along the sides of their bodies and their knees bent. To maintain a neutral position of the head and neck, a thin pillow (about 3–4 cm) was placed under the head [19,22]. Imaging of the LC muscle was obtained 2 cm below the thyroid cartilage on the anterior aspect of the neck, with the probe placed perpendicular to the skin. The thyroid cartilage was palpated, and a bilateral image was taken when the LC muscle was relaxed [19]. The LC muscle was clearly visible after placing the probe horizontally on the surface and tilting it upwards and downwards (Figure 2).

#### 2.4.2. Ultrasound Imaging Measurement

The lateral dimension (LD) of the muscle was calculated by measuring the longest distance between the medial and lateral borders of each muscle. The antero-posterior dimension (APD) was measured as the greatest depth between the ventral and dorsal boundaries of the muscles and was calculated on the middle of the LD [38,39].

To assess the cross-sectional area (CSA) of the LC, the borders of the muscle were recognized through the vertebral body (medially and inferiorly), carotid artery (superiorly and laterally), and retropharyngeal space (superiorly and medially) [22]. Moreover, the CSA of the CM was evaluated from the echogenic spinous process in the medial border, echogenic fascia between the cervical multifidus and semispinalis in the superior border, and echogenic fascia between the cervical multifidus and semispinalis in the inferior border [41].

In order to assess the EI features of the CM and LC, the images were transferred to offline DICOM ImageJ software version 1.53 (National Institutes of Health, Bethesda, MD, USA) and processed according to previous studies [33,42]. First, all RGB (Red–Green–Blue) images were converted into 256 gray-scale 32-bit images. The region of interest (ROI) was then selected in CM and LC muscles, avoiding surrounding bones and fascia. The EI in the ROI was presented as a value between 0 and 255 (0: black; 255: white).

#### 2.4.3. Clinical Features: Neck Pain Intensity and Related Disability

The intensity of neck pain was assessed with an 11-point numerical pain rate scale (NPRS, 0–10). Neck pain-related disability was assessed by using the Neck Disability Index (NDI), which is a reliable and valid self-rated tool for assessing neck pain disability consisting of 10 items related to pain intensity, headache, concentration, and various physical activities (lifting, driving, work, personal care, recreation, reading, and sleeping). In the NDI questionnaire, each item is scored from 0 to 5, with a final score of 0 to 100 [43].

### 2.5. Statistical Analyses

Statistical Package for the Social Sciences (SPSS v.29.1.1, Armonk, NY, USA) for Sonoma OS (Mac OS v.14.0) was used to perform statistical analyses, with a significance threshold of *p* < 0.05. The distribution of the data was assessed using histograms and the Shapiro–Wilk tests for continuous data points. If the *p* value was less than 0.05, it indicated a non-normal distribution, while a *p* value greater than 0.05 denoted a normal distribution. Descriptive statistics detailed the overall sample traits. For categorical data, the count and proportion for each group were presented (for instance, the count and proportion of females and males). For continuous data, measures of central tendency (mean for normally distributed variables and median for variables not normally distributed) were used, with their measures of spread (standard deviation for normally distributed data and interquartile range for data not following a normal distribution).

Between-group differences were assessed by using multivariate linear general models, including each US measure for both CM and LC muscles (CSA, AP dimension, lateral dimension and EI) as dependent variables, group (bulging, protrusion, extrusion, and controls), and side (symptomatic and asymptomatic in patients) as main factors and BMI, sex, and age as covariates. Since no side-to-side differences between the left and right sides in the control group were observed (*t*-test for independent samples), the mean value of both sides was used in the main analyses. As multiple comparisons were calculated, post hoc tests were conducted (group ×side) by using Bonferroni correction. The effect size was estimated using $\eta_{p}^{2}$ (a score of 0.01 was considered small, 0.06 was considered medium, and 0.14 was considered large). *p* values were assumed to be significant at <0.00625 (0.05/8) [44].

Finally, a Pearson’s correlation (r) matrix was calculated by selecting the three groups of patients and including demographic variables (BMI and age), clinical severity indicators (duration of symptoms, pain intensity, and related disability) and US features (CSA, APD, LD and MEI) for both CM and LC. This matrix analyzed the direction (positive values indicate proportionally directed association and negative values proportionally undirected association) and strength (absolute r values ranging between 0 and 0.3 were considered poor, between 0.3 and 0.6 were considered fair, 0.6 to 0.8 were considered moderate, and 0.8 to 1.0 were considered strong) of the paired correlations and was used to identify covariance and shared variance among the variables [45].

## 3. Results

### 3.1. Participants

Seventy-six consecutive patients were initially screened for possible adherence to the eligibility criteria. Finally, a total of sixty (*n* = 60) patients satisfied all the eligibility criteria and agreed to participate. All of the excluded participants declined their participation in the later data collection for personal reasons (*n* = 16). Fifteen patients exhibited cervical disc bulging (20% male; mean age: 48.5; SD, 7.5 years), fifteen exhibited cervical disc protrusion (6% male; mean age: 43; SD, 7.8 years), and fifteen exhibited cervical disc extrusion (40% male; mean age: 44; SD, 8 years). Additionally, fifteen asymptomatic controls (40% male; mean age: 40.4; SD, 9.7 years) were also included. Table 1 summarizes the clinical data of the patient groups. Due to the differences (although not significant) in age, sex distribution, and BMI among the four groups, these variables were included as covariates. Patients with disc extrusion exhibited higher related disability (NDI score) than the disc bulging (*p* < 0.001) and disc protrusion (*p* = 0.006) groups. Further, the disc extrusion group also showed higher pain intensity (NPRS) than the disc bulging group (*p* = 0.002).

### 3.2. Ultrasound Measurements of the Cervical Multifidus

The multivariate linear general model did not reveal any significant group or side effect or group ×side interaction for CSA and LD of the CM. A significant group, but not side or group ×side interaction, effect for APD and MEI of the CM was found. The post hoc analyses revealed that patients with cervical disc extrusion exhibited bilateral lower APD of the CM than healthy controls (*p* = 0.006) but similar APD to those with disc bulging (*p* = 0.07) or disc protrusion (*p* = 0.567). Additionally, patients with cervical disc protrusion (*p* = 0.004) or disc extrusion (*p* = 0.001) exhibited significantly higher CM mean brightness compared with healthy controls but similar to those with disc bulging (*p* = 0.545). Table 2 details US measurements of the CM in patients with cervical disc bulging, disc protrusion, disc extrusion, and the asymptomatic controls.

### 3.3. Ultrasound Measurements of the Longus Colli

Table 2 details the US measurements of the LM in patients with cervical disc bulging, disc protrusion or disc extrusion as well as asymptomatic controls. The multivariate linear general model revealed significant group, but not side or group ×side interaction, effect for CSA, APD and MEI of the LC muscle. No significant group/side effect or group ×side interaction for LD was observed. The post hoc analysis revealed that individuals with cervical disc protrusion or disc extrusion exhibited significantly lower CSA of the LC than the controls (*p* < 0.001) and those with disc bulging (*p* = 0.004). There was a tendency for a lower CSA of the LC in the symptomatic side both cervical disc protrusion and extrusion groups but it did not reach statistical significance (Table 2). Patients with cervical disc protrusion or extrusion exhibited significantly lower APD of the LC when compared with healthy controls (*p* < 0.001) and with those with disc bulging (*p* = 0.004). The symptomatic side of patients with cervical disc extrusion showed the lowest APD of the LC, although differences were not significant (Table 2).

### 3.4. Effects of Covariates in Ultrasound Measurements

The inclusion of the covariates revealed a significant effect of sex for CSA and MEI of the LC (CSA: F = 7.680, *p* = 0.007, $\eta_{p}^{2}$ = 0.066; APD: F = 4.611, *p* = 0.034, $\eta_{p}^{2}$ = 0.041; LD: F = 0.604, *p* = 0.439, $\eta_{p}^{2}$ = 0.006; MEI: F = 9.110, *p* = 0.003, $\eta_{p}^{2}$ = 0.077) and for all US measurements of the CM (CSA: F = 41.107, *p* < 0.001, $\eta_{p}^{2}$ = 0.274; APD: F = 16.161, *p* < 0.001, $\eta_{p}^{2}$ = 0.129; LD: F = 13.210, *p* < 0.001, $\eta_{p}^{2}$ = 0.108; MEI: F = 22.835, *p* < 0.001, $\eta_{p}^{2}$ = 0.173): Females showed lower CSA and lower brightness of the LC; and lower CSA, smaller APD and LD length, and lower brightness of the CM muscle when compared with males.

No significant effect of age for any US measurement of the LC (CSA: F = 5.928, *p* = 0.017, $\eta_{p}^{2}$ = 0.052; APD: F = 2.008, *p* = 0.159, $\eta_{p}^{2}$ = 0.018; LD: F = 6.690, *p* = 0.011, $\eta_{p}^{2}$ = 0.058; MEI: F = 1.039, *p* = 0.310, $\eta_{p}^{2}$ = 0.009) or CM (CSA: F = 6.460, *p* = 0.012, $\eta_{p}^{2}$ = 0.056; APD: F = 4.852, *p* = 0.030, $\eta_{p}^{2}$ = 0.043; LD: F = 5.521, *p* = 0.024, $\eta_{p}^{2}$ = 0.046; MEI: F = 6.130, *p* = 0.015, $\eta_{p}^{2}$ = 0.053) was identified.

No significant effect of BMI for the LC was found (CSA: F = 0.221, *p* = 0.822, $\eta_{p}^{2}$ = 0.001; APD: F = 2.797, *p* = 0.097, $\eta_{p}^{2}$ = 0.025; LD: F = 0.445, *p* = 0.506, $\eta_{p}^{2}$ = 0.004; MEI: F = 2.990, *p* = 0.087, $\eta_{p}^{2}$ = 0.027). However, a significant effect of BMI for most US measurements of the CM was identified (CSA: F = 22.218, *p* < 0.001, $\eta_{p}^{2}$ = 0.169; APD: F = 7.143, *p* = 0.009, $\eta_{p}^{2}$ = 0.062; LD: F = 4.167, *p* = 0.044, $\eta_{p}^{2}$ = 0.037; MEI: F = 13.864, *p* < 0.001, $\eta_{p}^{2}$ = 0.113).

### 3.5. Correlation Analysis

The multivariate correlation matrix is represented in Table 3. Although none of the US variables were associated with pain duration (all, *p* > 0.05), some of the US metrics were associated with related disability and pain intensity. For instance, the minor axis (LD) of the LC muscle and the CM major and minor axes (APD and LD) were negatively associated with related disability (*p* < 0.01; *p* < 0.05 and *p* < 0.01, respectively) and pain intensity was negatively associated with LC major and minor axes (both, *p* < 0.05).

## 4. Discussion

To the best of the author’s knowledge, this is the first study to investigate muscle morphology and brightness differences in the CM and LC muscles in individuals with unilateral cervical herniated discs. The most important findings were that patients exhibited bilateral decreased CSA and differences in muscle shape when compared with pain-free controls. In addition, although no significant differences among the three subgroups of patients with cervical disc (bulging, protrusion or extrusion) were identified for the CM, patients with cervical disc protrusion or extrusion were characterized by lower CSA and APD in comparison with patients with disc bulging. Finally, we found US morphological metrics to be associated with pain intensity and related disability but not with the duration of the symptoms (chronicity).

The main rationale for conducting this study was that, although neck pain symptoms can be associated with degenerative processes or anatomical alterations observed in radiological images, there is an absence of correlation between imaging findings and the clinical situation in most patients suffering neck pain (but serious conditions, such as cervical myelopathy, cervical ligamentous instability, fracture, neoplasm or vascular insufficiency) [12]. Consequently, current recommendations provided by clinical practice guidelines for the assessment of patients with neck pain include the assessment of the function of muscles and nerves, as these structures could also be sources of nociception [12].

Although herniated cervical discs are commonly associated with cervical myelopathies, the radiological images are not clearly associated with their clinical presentation [46]. Therefore, even if there are available guidelines for the use of radiological imaging procedures for these patients [47], its prognostic value remains controversial and depends on the current classification of individuals with neck pain (neck pain associated with mobility deficits, radiating pain, motor control impairments, and headache). For instance, while patients referring neurological signs, with non-resolving radiculopathy, progressing myelopathy, or suffering a motor vehicle crash are encouraged to undergo radiological exams, patients with mobility deficits (either in an acute or chronic stage) in the absence of red flags are exempt from these imaging procedures [12].

A single study (using MRI) assessed the association among cervical paraspinal muscle morphological and histological changes, degeneration of cervical joint structures, and clinical outcomes in patients with chronic non-specific neck pain [48]. This study showed significant correlations between the percentage of fatty infiltration and muscle atrophy with the grade of disc degeneration in specific muscle groups. For instance, at the C3/C4 level, there was a positive correlation between the fatty infiltration percentage of certain musculature (e.g., the semispinalis cervicis and capitis, splenius cervicis and capitis, levator scapulae and, bilaterally, LC and longus capitis) and the grade of disc degeneration at the same level. These findings are in accordance with our results since the increased MEI observed at the LC and CM in the cohort of patients with neck pain in comparison with the control group could reflect this fatty infiltration shown through the use of MRI. Additionally, morphological changes in deep extensor muscles and superficial paraspinal muscles were strongly linked with cervical balance parameters. In terms of clinical features, the study found that pain-related fatty infiltration changes were predominantly observed unilaterally (right side) in extensor muscles. Thus, the authors described multiple associations among histological and morphological muscle changes, cervical spine degeneration, and the clinical progression of neck pain. The results obtained in this study using US are consistent with the study using MRI, as we found multiple associations among muscle size and morphology with clinical indicators.

These findings are contrary to our results, where bilateral changes in the CM and LC were identified. Although both studies analyzed samples with similar demographic characteristics, no direct comparisons can be carried out as there were critical sample issues between both studies. For instance, these issues concerned the differences regarding the instruments used for assessing neck pain-related disability (Northwick Park Neck Pain Questionnaire versus Neck Disability Index) and a lack of details about clinical information in both studies (no pain duration information was provided by Huang et al., and no recurrence of acute episodes or biomechanical characteristics were considered in this research). However, the differences in the results could be explained by pain intensity differences between the samples. Our sample reported greater pain intensity (5.3 to 6.8 depending on the groups versus 4.35 in the sample analyzed by Huang et al. [48]). This hypothesis could be reinforced as Javanshir et al. [19] also found bilateral LC atrophy using US in a sample with chronic neck pain and a similar pain intensity (NPRS, 5.1).

### Limitations

Although the strengths of this project have been highlighted, some limitations need to be recognized. The most important limitation is that the controls did not undergo any radiological exam to confirm the absence of disc lesions [49,50]. Therefore, future studies with more resources should include an MRI exam for all the participants to control the asymptomatic radiological findings factor. In addition, only patients with unilateral neck pain were assessed, and these conclusions may not be extrapolated to populations with bilateral neck radicular pain. Additionally, it should be considered that further studies include samples with wider ranges of pain duration, disability, pain intensity, and demographic characteristics, which may result in more supported conclusions. Finally, this study only calculated the MEI as a metric of muscle quality. Despite the fact that the evidence described this procedure as reliable, calculating the intramuscular fat percentage may provide more accurate results.

## 5. Conclusions

The findings of our study indicate significant differences in morphology and echogenicity of the CM and LC muscles among patients with varying degrees of cervical disc herniation and asymptomatic controls. Regarding the CM muscle, patients with cervical disc extrusion demonstrated a bilateral lower APD compared to the healthy controls, which was similar to those with disc bulging or protrusion, whereas patients with cervical disc protrusion or extrusion exhibited significantly higher mean echogenicity compared to the healthy controls. This suggests increased echogenicity in these groups, potentially indicating changes in muscle composition.

Regarding the LC findings, patients with cervical disc protrusion or extrusion showed significantly lower CSA and APD of the LC compared to the healthy controls and those with disc bulging. Changes in these muscles were also correlated with higher pain intensity and disability. Future research with more diverse samples and additional metrics of muscle quality, such as intramuscular fat percentage, is recommended to support and expand upon these findings.

## Figures and Tables

**Table 1:** Demographic and clinical features of participants.

	Controls	Disc Bulging	Disc Protrusion	Disc Extrusion	*p*-Value
Age(year)	40.4 ± 9.7	48.5 ± 7.5	43.1 ± 8.0	43.9 ± 8.0	0.07
BMI (kg/m^2^)	26 ± 3.9	29.2 ± 6.4	26.7 ± 3.6	27.8 ± 4.8	0.29
Sex (female/male)	9/6	12/3	14/1	9/6	0.1
Dominant side (Right/left)	15/0	14/1	15/0	13/2	0.28
Pain side (Left/Right)	-	6/9	7/8	7/8	0.91
NDI (0–100) ^†^	-	28.4 ± 7.6	33.9 ± 7.8	45.2 ± 12.1	<0.001
Pain duration (months)	-	8.6 ± 4.5	10.7 ± 8.1	6.5 ± 3.3	0.14
NPRS (0–10) ^†^	-	5.3 ± 1.2	5.9 ± 1.2	6.8 ± 1.1	0.003

BMI: body mass index; NDI: neck disability index; NPRS: numerical pain rate scale; ^†^ statistically significant differences between the groups.

**Table 2:** Ultrasound (US) measurements of the cervical multifidus (CM) and longus colli (LC) muscles.

Group	Side	Cervical Multifidus				Longus Colli			
		APD	LD	CSA	MEI	APD	LD	CSA	MEI
Baseline Scores									
Disc Bulging	Non-symptomatic	6.8 ± 1.0	23.4 ± 2.6	120.8 ± 21.6	60.9 ± 12.5	7.5 ± 1.1	12.5 ± 1.1	80.3 ± 8.4	79.7 ± 10.4
	Symptomatic	6.5 ± 0.9	23.9 ± 2.3	117.7 ± 19.3	62.8 ± 14.1	7.5 ± 0.7	12.1 ± 1.4	78.9 ± 9.2	82.3 ± 11.9
Disc Protrusion	Non-symptomatic	6.3 ± 0.4	23.5 ± 2.5	117.4 ± 15.2	65.5 ± 9.8	6.8 ± 0.5	12.1 ± 1.6	75.2 ± 8.3	72.3 ± 7.4
	Symptomatic	6.1 ± 0.5	22.9 ± 2.4	107.0 ± 15.3	65.9 ± 12.2	6.5 ± 0.5	11.6 ± 0.9	68.6 ± 6.4	77.7 ± 6.0
Disc Extrusion	Non-symptomatic	6.3 ± 0.8	22.5 ± 1.9	113.4 ± 14.9	63.7 ± 15.0	7.0 ± 0.8	11.7 ± 1.6	71.8 ± 8.7	85.2 ± 10.1
	Symptomatic	5.8 ± 0.7	22.0 ± 1.7	109.4 ± 11.1	65.6 ± 12.3	6.5 ± 0.5	11.6 ± 0.9	68.6 ± 6.4	87.5 ± 12.4
Controls	Non-symptomatic (Right)	6.6 ± 0.2	23.1 ± 0.5	120.5 ± 4.1	57.0 ± 1.9	7.5 ± 0.2	12.1 ± 0.4	80.3 ± 2.2	72.1 ± 2.4
	Non-symptomatic (Left)	6.7 ± 0.2	23.3 ± 0.5	121.1 ± 4.2	56.5 ± 1.9	7.3 ± 0.2	12.2 ± 0.4	79.4 ± 2.2	72.4 ± 2.4
Differences Analysis									
Group	F	4.408	2.441	1.215	9.292	8461	1.091	9.954	16.808
	*p* Value	0.006	0.068	0.308	<0.001	<0.001	0.356	<0.001	<0.001
	$\eta_{p}^{2}$	0.108	0.063	0.032	0.204	0.189	0.029	0.215	0.316
Side	F	5.186	0.151	3.228	0.360	2.582	1.153	5.467	2.365
	*p* Value	0.025	0.698	0.075	0.499	0.111	0.285	0.021	0.127
	$\eta_{p}^{2}$	0.045	0.001	0.029	0.003	0.023	0.010	0.048	0.021
Group ×Side	F	0.784	0.501	0.795	0.083	0.887	0.278	1.213	0.435
	*p* Value	0.505	0.682	0.499	0.969	0.451	0.841	0.308	0.728
	$\eta_{p}^{2}$	0.021	0.014	0.021	0.002	0.024	0.008	0.032	0.012

APD: anterior posterior dimension (mm); LD: lateral dimension (mm; CSA: cross-sectional area (mm^2^); MEI: mean echo intensity (0–255).

**Table 3:** Pearson’s correlation matrix for assessing the association between US characteristics and pain-related indicators.

	1	2	3	4	5	6	7	8	9	10
1. Neck Disability Index										
2. Pain Intensity	0.448 **									
3. Pain Duration	−0.366 **	n.s.								
4. Longus Colli—CSA	n.s.	n.s.	n.s.							
5. Longus Colli—APD	−0.386 **	−0.237 *	n.s.	0.530 **						
6. Longus Colli—LD	n.s.	−0.225 *	n.s.	0.548 **	n.s.					
7. Longus Colli—MEI	n.s.	n.s.	n.s.	−0.269 **	n.s.	n.s.				
8. Cervical Multifidus—CSA	n.s.	n.s.	n.s.	0.355 **	0.418 **	n.s.	n.s.			
9. Cervical Multifidus—APD	−0.217 *	n.s.	n.s.	0.319 **	0.342 **	n.s.	n.s.	0.664 **		
10. Cervical Multifidus—LD	−0.316 **	n.s.	n.s.	0.262 **	0.408 **	n.s.	n.s.	0.619 **	n.s.	
11. Cervical Multifidus—MEI	n.s.	n.s.	n.s.	n.s.	n.s.	n.s.	0.326 **	n.s.	n.s.	n.s.

* Statistically significant differences (*p* < 0.05); ** Statistically significant differences (*p* < 0.01); n.s. non-significant association.

## Data Availability

The authors confirm that the data supporting the findings of this study are available within the article.
